# Identification of disease-specific gut microbial markers in vitiligo

**DOI:** 10.3389/fmicb.2025.1499035

**Published:** 2025-02-04

**Authors:** Yimin Dou, Yi Niu, Hexiao Shen, Lan Wang, Yongling Lv, Suwen Liu, Xiafei Xie, Aiping Feng, Xinxin Liu

**Affiliations:** ^1^Department of Dermatology, Union Hospital, Tongji Medical College, Huazhong University of Science and Technology, Wuhan, China; ^2^Department of Gastroenterology Surgery, Union Hospital, Tongji Medical College, Huazhong University of Science and Technology, Wuhan, China; ^3^School of Life Science, Hubei University, Wuhan, China

**Keywords:** vitiligo, gut microbiome, gut-skin axis, 16S rRNA sequence, VASI

## Abstract

There is a potential correlation between vitiligo and gut microbiota, although research in this area is currently limited. The research employed high-throughput sequencing of 16S rRNA to examine the gut microbiome in the stool samples of 49 individuals with vitiligo and 49 without the condition. The study encompassed four comparison groups: (1) DI (disease) group vs. HC (healthy control) group; (2) DI_m group (disease group of minors) vs. HC_m group (healthy control group of minors); (3) DI_a group (adult disease group) vs. HC_a group (adult healthy control group); (4) DI_m group vs. DI_a group. Research findings have indicated the presence of spatial heterogeneity in the gut microbiota composition between individuals with vitiligo and healthy controls. A significant reduction in gut microbiota diversity has been observed in vitiligo patients across both minors and adult groups. However, variations have been noted in the composition of disease-related differential microbial markers among different age groups. Specifically, *Bacteroides* and *Parabacteroides* have been identified as specific markers of the intestinal microbiota of vitiligo patients in both minor and adult groups. Correlative analyses have revealed a positive correlation of these two genera with the Vitiligo Area Scoring Index (VASI) and disease duration. It is noteworthy that there are no significant differences in diversity between the DI_m group and the DI_a group, with similarities in microbiota composition and functional characteristics. Nevertheless, correlative analyses suggest a declining trend in *Bacteroides* and *Parabacteroides* with increasing age. Individuals with vitiligo exhibit distinct features in their gut microbiome when contrasted with those in the healthy control group. Additionally, the microbial marker genera that show variances between patients and healthy controls vary among different age groups. Disease-specific microbial marker genera (*Bacteroides* and *Parabacteroides*) are associated with VASI, duration of the condition, and age. These findings are essential for improving early diagnosis and developing potential treatment strategies for individuals with vitiligo.

## Introduction

Vitiligo is a widespread condition affecting skin pigmentation, impacting around 0.5 to 2% of people worldwide (Bergqvist and Ezzedine, 2020). Presently, it is classified as an autoimmune disorder, with its development linked to the excessive activation of CD8+ T cells, which are self-reactive cells located in the skin (Perez-Bootello et al., 2023). Overactivation of CD8+ T cells results in the release of IFN-*γ*. IFN-γ at the diseased skin can increase the expression of keratinocyte chemokines such as CXCL9 and CXCL10, which subsequently attract effector T cells that attack melanocytes (Perez-Bootello et al., 2023; Lee et al., 2024). Recent studies indicate that gut bacteria are essential in forming and sustaining the human innate immune system (Zheng et al., 2020; Donald and Finlay, 2023). The gut microbiome plays a crucial role in immune-related diseases like rheumatoid arthritis, systemic lupus erythematosus, and autoimmune pancreatitis, and is also closely linked to several skin conditions (Ruff et al., 2020; Colucci and Moretti, 2021).

The complex interplay among the gut microbiota-immune-skin axis has driven research into skin conditions linked to the gut microbiota (Mahmud et al., 2022). Besides prevalent conditions such as eczema and psoriasis, research on the connection between gut bacteria and vitiligo has underscored the crucial influence of gut microbiota on immune-related skin diseases (Chen et al., 2021b). Dellacecca et al.’s (2020) animal studies have shown that an imbalance in the gut microbiota, induced by antibiotics, can trigger the development of vitiligo in mice. Research involving humans has shown that people with vitiligo exhibit reduced gut microbiota richness and diversity compared to those without the condition. Additionally, there are marked alterations in species composition, including a notable rise in the *Firmicutes/Bacteroidetes* ratio among vitiligo sufferers (Bzioueche et al., 2021; Wu et al., 2023). Furthermore, Ni et al. (2020) combined microbiome sequencing with serum untargeted metabolomics analysis to establish a strong correlation between specific gut microbiota genera, the progression of vitiligo, and serum IL-1β levels. Although these discoveries hint at a possible connection between gut bacteria and vitiligo, the scarcity of current research makes the matter complex, particularly due to the lack of studies considering age-related aspects. Therefore, further research is essential to clarify the impact of age on gut microbiota in relation to vitiligo.

This research thoroughly examined the species makeup and possible roles of gut microbiota in individuals with vitiligo and healthy subjects through 16S rRNA high-throughput sequencing. To evaluate how age affects the gut microbiota composition in individuals with vitiligo, the participants were categorized by age. The results not only expand our understanding of the gut microbiome’s connection to vitiligo but also reveal, for the first time, age-specific differences in gut microbiome markers among vitiligo sufferers. These findings enhance our comprehension of how gut microbiota may influence vitiligo development and provide valuable insights for therapeutic approaches that involve altering gut microbiota.

## Materials and methods

### Participant recruitment

The research participants consisted of individuals diagnosed with vitiligo and healthy individuals selected from the Department of Dermatology at Union Hospital Affiliated with Huazhong University of Science and Technology. The research received authorization from the hospital’s Medical Ethics Committee [[2019] Lun Shen Zi (S048)], and participants or their legal representatives were informed and consented to join the study. This study primarily focuses on patients with stable non-segmental vitiligo. The diagnosis of the disease stage and type is based on consensus (Lei et al., 2021b). The following inclusion criteria were used for vitiligo patients: (1) Non-segmental vitiligo; (2) Vitiligo Disease Activity (VIDA) score of 0; (3) Clinical characteristics: white lesions with clear edges or pigmentation; (4) No isomorphic reaction (≥ 1 year); (5) Wood lamp: white lesions with clear edges, and the lesion area under the Wood lamp ≤ visual area; (6) No family genetic history of vitiligo. The participants were categorized into two groups: a minor group (aged 6–18 years) and an adult group (aged 18–60 years). We also enlisted healthy individuals who were comparable in terms of gender, age, and body mass index (BMI). Participants were excluded if they had taken antibiotics or immunosuppressive drugs in the 2 weeks before sampling, or if they had any other illnesses such as autoimmune disorders, metabolic conditions, cancers, or organ failure, or had been exposed to chemical triggers (e.g., monobenzyl ether of hydroquinone, butylphenol, phenol, tert-butylphenol, pyrocatechol). Pregnant or lactating women were also excluded. Demographic data such as age, gender, BMI, and dietary patterns were collected from all participants through structured questionnaires.

### Sample collection, DNA extraction, and 16S rRNA sequencing

Participants gathered all stool specimens at home using sterile collection kits, promptly transported them to the hospital on dry ice, and stored them at −80°C until further analysis. According to the instructions of HiPure Stool DNA Mini Kit (Magen, Guangzhou, China), 200 mg of stool sample was taken and lysis buffer was added to promote cell rupture and DNA release. After a series of centrifugation steps to remove cellular debris and impurities, the DNA was collected in the supernatant. Then binding buffer was added to bind the DNA to the columns in the kit, and impurities were removed by a washing step, and finally the pure DNA was collected by elution buffer. Afterward, the amount of microbial DNA was measured with a Qubit device, and its quality was evaluated through 1% agarose gel electrophoresis. DNA concentration ≥ 20 ng/μL and volume ≥ 20 μL (total amount ≥ 400 ng) were used as quality control criteria. In this study, the sample DNA concentration ranged from 35.09–82.36 ng/μL and the total amount ranged from 2807.2 to 6588.8 ng. To ensure the accuracy and comparability of the subsequent experiments, we homogenized all the samples in the PCR step of amplifying the 16S rRNA gene, strictly controlling the total amount of DNA used for amplification in each sample to be consistent, so as to avoid the influence on the subsequent experiments due to the difference in the initial DNA concentration. The V3-V4 region of the 16S rRNA gene was amplified by PCR using the primers 341F (5’-CCTACGGGNGGCWGCAG-3′) and 805R (5’-GACTACHVGGGTATCTAATCC-3′). After PCR amplification, the product was purified using magnetic beads, its concentration was determined with Qubit, and the size was confirmed by 1.5% agarose gel electrophoresis (Biowest Agarose was used for agarose gel and Tris–HCl for buffer). Afterward, the FS DNA Lib Prep Kit V6 (Abclonal, Wuhan, China) was employed for library construction, and its concentration was measured using Qubit. After a positive evaluation, the library underwent sequencing on an Illumina Miseq device utilizing the PE250 technique.

### Microbiome analysis

The methodology comprises multiple steps, including initial filtering of R1 and R2 reads, exclusion of reads containing N, and elimination of reads with estimated errors exceeding 2. Base quality evaluation is performed from the 5′ end to the 3′ end. If the base quality drops below 2, all bases from that point to the 3′ end are removed. Error estimation models are constructed separately for filtered R1 and R2 reads. Amplicon Sequence Variants (ASV) are deduced based on the error estimation models of R1 and R2. Denoised R1 and R2 sequences are merged, and chimeras are removed using the *de novo* method integrated into DADA2. Subsequent to the elimination of erroneous sequences, a 100% identity threshold is employed for clustering, and ASV feature sequences are segregated.

The feature-classifier classify-sklearn in QIIME2 is used with the SILVA database (v. 138.2) for taxonomic annotation of feature sequences. The Microeco package in R is used to analyze *α* diversity metrics, such as the ACE, Chao, Shannon, and Simpson indices. *β* diversity was assessed using the Jaccard, Bray-Curtis, weighted UniFrac, and unweighted UniFrac distance metrics. LEfse (Linear Discriminant Analysis Effect Size, LDA Effect Size) is employed to identify microbial biomarkers that show notable differences between two groups. Functional prediction of the microbiota is conducted using PICRUST2 software. The ggplot2 package in R is used for visualization.

### Statistical analysis

To evaluate significant differences between the two groups for continuous variables like age, BMI, VASI, and disease duration, a T-test was utilized. For categorical variables such as gender, the Kruskal-Wallis test was employed to examine the differences among the groups. Two-tailed Wilcoxon rank-sum tests and PERMANOVA were employed to calculate the *p*-values for *α*-diversity and *β*-diversity, respectively. The Wilcoxon rank sum test in R software was utilized to assess the variations in the relative proportions of gut microbiota between the two groups. In the LEfSe analysis, microorganisms with an LDA score > 2.0 were identified as species exhibiting significant differences between groups. The lm function in R software was utilized to conduct a linear correlation analysis. *p*-values corrected using the Benjamini-Hochberg method were deemed statistically significant if they were below 0.05.

## Results

### Characteristics of the study population

Individual characteristics, such as age, gender, BMI, and dietary habits of the participants, were gathered through a questionnaire survey. In the case of vitiligo patients, additional data on disease classification, disease activity level, disease severity, and disease duration were also collected. The study comprised 98 participants who met the specified inclusion and exclusion criteria. This comprised 49 non-segmental vitiligo patients diagnosed in the stable phase and 49 healthy individuals, who were, respectively, allocated to the DI group (disease group) and HC group (healthy control group). The two groups showed no notable differences in terms of gender distribution, age, BMI, and dietary habits, with both following omnivorous diets. Moreover, the groups were stratified by age, with the DI group further categorized into DI_m group (disease group of minors, ages 3–18, 18 males and 5 females) and DI_a group (disease group of adults, ages 19–60, 15 males and 11 females), and the HC group divided into HC_m group (healthy control group of minors, ages 3–18, 17 males and 7 females) and HC_a group (healthy control group of adults, ages 19–60, 15 males and 10 females). The differences in population characteristics between the DI_m group and DI_a group, DI_m group and HC_m group, DI_a group and HC_a group, as well as HC_m group and HC_a group were analyzed separately. The variances in population characteristics among distinct age subgroups (DI_m group and HC_m group; DI_a group and HC_a group) were examined individually. Likewise, there were no notable variations in age, BMI, or gender distribution. The VASI scores and disease duration showed no notable differences between the DI_m and DI_a groups. Except for the notable age disparity, the two groups showed no major differences in BMI and gender distribution (Table 1).

**Table 1:** Characteristics of the study population.

Characteristic	DI (*n* = 49)	HC (*n* = 49)	*p*-value	DI_m (n = 23)	HC_m (n = 24)	*p*-value	DI_a (n = 26)	HC_a (n = 25)	*p*-value	DI_m (n = 23)	DI_a (n = 26)	*p*-value	HC_m (n = 24)	HC_a (n = 25)	*p*-value
Age, year, mean ± SD	24.94 ± 16.0	25.31 ± 15.01	0.907	11.17 ± 3.61	11.67 ± 2.93	0.611	37.12 ± 12.31	38.40 ± 8.83	0.67	11.17 ± 3.61	37.12 ± 12.31	<0.0001	11.67 ± 2.93	38.40 ± 8.83	<0.0001
BMI, kg/m^2^, mean ± SD	20.55 ± 3.70	20.71 ± 3.34	0.816	19.55 ± 4.03	18.92 ± 2.77	0.541	21.43 ± 3.21	22.43 ± 2.96	0.252	19.55 ± 4.03	21.43 ± 3.21	0.0795	18.92 ± 2.77	22.43 ± 2.96	<0.0001
Gender, male/female	33/16	32/17	0.832	18/5	17/7	0.564	15/11	15/10	0.868	18/5	15/11	0.129	17/7	15/10	0.438
VASI, mean ± SD	10.20 ± 5.74	–	–	10.00 ± 5.30	–	–	10.38 ± 6.20	–	–	10.00 ± 5.30	10.38 ± 6.20	0.819	–	–	–
Duration of disease, month	17.63 ± 9.44	–	–	16.17 ± 7.68	–	–	18.92 ± 10.76	–	–	16.17 ± 7.68	18.92 ± 10.76	0.305	–	–	–
Smoking, *n*	8	4	–	1	0	–	7	4	–	1	7	–	0	4	–
Alcohol consumption, *n*	11	7	–	0	0	–	11	7	–	0	11	–	0	7	–
race															
Han nationality	49	49	–	23	24	–	26	25	–	23	26	–	24	25	–
Educational level															
Elementary school	9	5	–	3	2	–	6	3	–	3	6	–	2	3	–
Junior high school	27	22	–	17	16	–	10	6	–	17	10	–	16	6	–
Senior high school	9	15	–	3	5	–	6	10	–	3	6	–	5	10	–
University	4	7	–	0	1	–	4	6	–	0	4	–	1	6	–
Marital status															
Single				23	24	–	3	5	–	23	3	–	24	5	–
Married				0	0	–	22	20	–	0	22	–	0	20	–
Divorced				0	0	–	1	0	-	0	1	–	0	0	–
Widowed				0	0	–	0	0	–	0	0	–	0	0	–

### Unique characteristics of gut microbiota in patients with vitiligo

**Figure 1:**
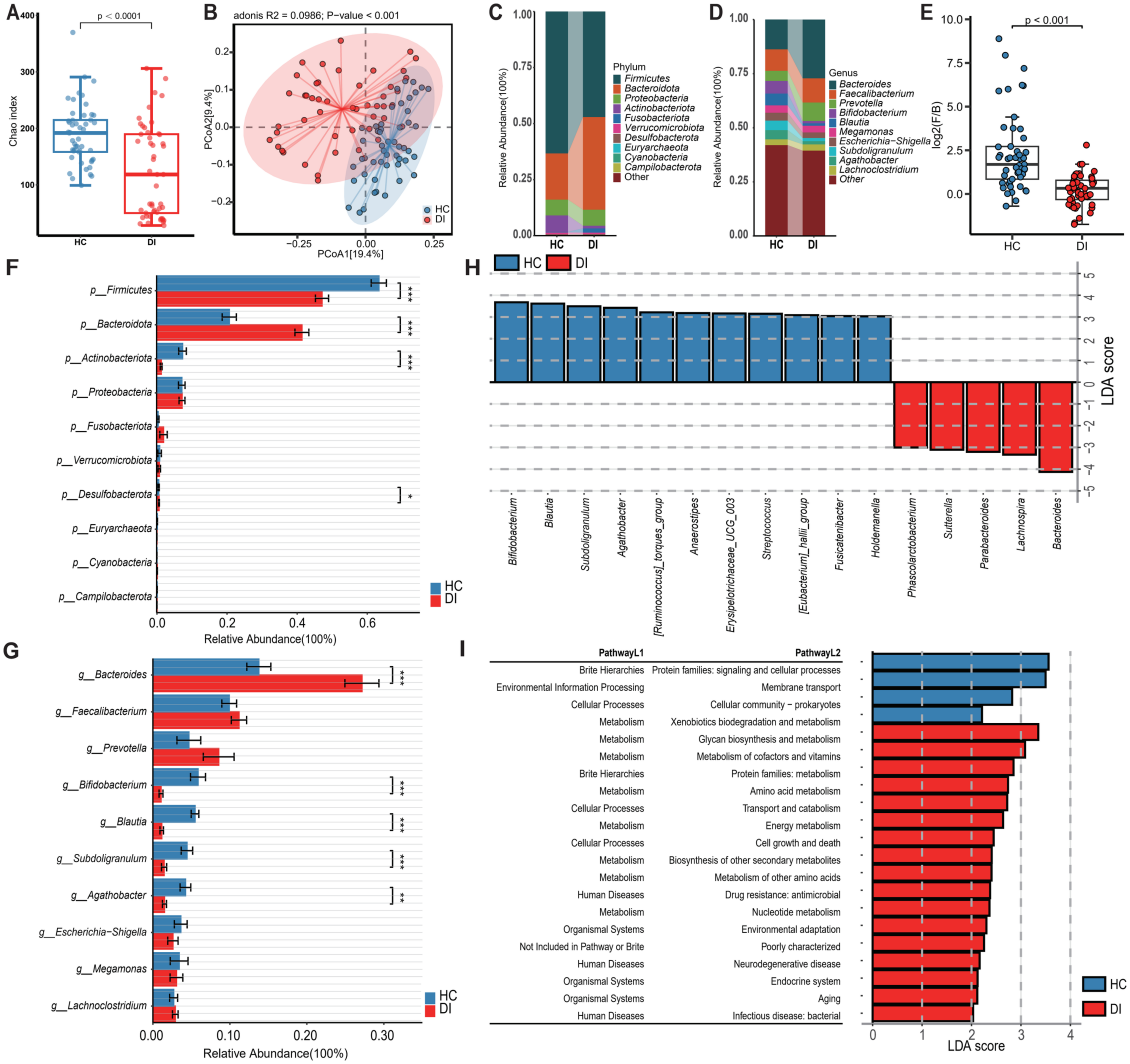
Characteristics of the gut microbiota in patients with vitiligo (DI) compared to healthy controls (HC). **(A)** The box scatter plot displays the Chao index of the two groups; **(B)** Principal coordinate analysis (PCoA) plot is based on unweighted_unifrac distance; **(C,D)** The histograms present the composition of the top 10 intestinal microbiota at the phylum level and genus level in the two groups; **(E)** The box scatter plot demonstrates the difference in the ratio of Firmicutes/Bacteroidetes; **(F,G)** The inter-group differences in the average relative abundance of the top 10 taxa at the phylum level and genus level are depicted. FDR-adjusted *p*-values are indicated as follows: *p < 0.05; **p < 0.01; ***p < 0.001; **(H)** LEfSe analysis at the genus level reveals the distinct marker genera of the intestinal tract between vitiligo patients and healthy controls (LDA score > 3); **(I)** LEfSe analysis based on KEGG level 1 and level 2 functional prediction pathways (LDA score > 2).

To understand the traits of gut microbiota in individuals with vitiligo, a preliminary comparison was conducted between the DI group and the HC group. The examination utilizing the Chao index revealed that the *α* diversity of the DI group was notably lower compared to the HC group (Figure 1A, *p* < 0.0001). Moreover, the *β* diversity, as determined by the Unweighted UniFrac distance, revealed a spatial heterogeneity in the composition of gut microbiota between the group with DI and the group of healthy controls (Figure 1B, *p* < 0.001). In summary, these findings indicate a significant diversity distinction in the gut microbiota between vitiligo patients and healthy controls.

The study conducted a detailed analysis of the variations in gut microbiota composition between the two groups under investigation. *Firmicutes*, *Bacteroidota*, and *Proteobacteria* were recognized as the main phyla at the phylum level, together making up more than 90% of the microbiota (Figures 1C,D and Supplementary Table S1). Remarkably, there were substantial variations in the levels of *Firmicutes* and *Bacteroidota* between the two groups, with *Bacteroidota* being notably enriched in the DI group and *Firmicutes* relatively more abundant in the HC group. As a result, the ratio of *Firmicutes* to *Bacteroidota* (F/B) was markedly reduced in the DI group relative to the HC group (Figure 1E, *p* < 0.0001). Furthermore, *Actinobacteriota* exhibited a significant enrichment in the HC group (Figure 1F). At the genus level, *Bacteroides* was the most dominant genus in both groups, however, its relative abundance was notably greater in the DI group than in the HC group.

In the HC group, *Bifidobacterium, Blautia, Subdoligranulum*, and *Agathobacter* were significantly more abundant among the top 10 genera by relative abundance (Figure 1G). Additionally, the LEfSe analysis revealed significant enrichment of *Bacteroides, Lachnospira, Parabacteroides, Sutterella,* and *Phascolarctobacterium* in the DI group, with *Bacteroides* exhibiting the highest LDA score (LDA_score = 4.31). In contrast, 11 genera including *Bifidobacterium, Blautia, Subdoligranulum* etc. were identified as specific gut microbiota markers in the HC group (Figure 1H). To further investigate the potential functional variances of gut microbiota, we utilized PICRUSt2 and the KEGG database to predict the functions of gut microbiota in all samples. Subsequently, we employed LEfSe analysis to identify function pathways with notable variances between the two groups (Figure 1I and Supplementary Table S2). Our analysis revealed four functional pathways with significant differences at KEGG level 2. In particular, the HC group had a higher prevalence of protein families related to signaling, cellular activities, and membrane transport, while the DI group showed significant enrichment in glycan biosynthesis, metabolism, and the processing of cofactors and vitamins. In addition, the level of HIF-1 signalling pathway enrichment was significantly lower in the DI group compared to the HC group (Supplementary Figures S2A–C).

### Gut microbial biomarkers associated with vitiligo demonstrate variations across different age groups

**Figure 2:**
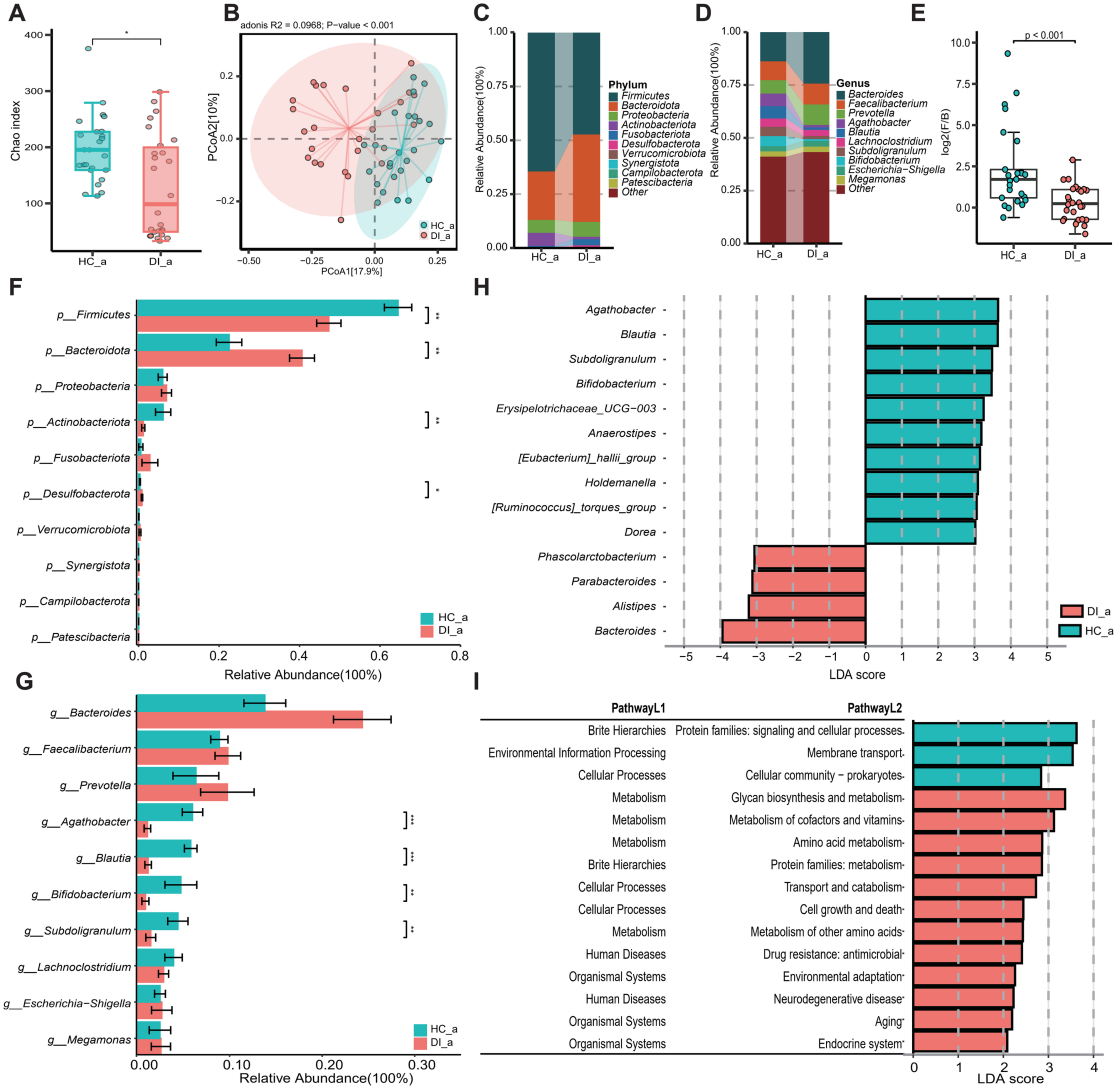
Subgroup1: Comparison of the gut microbiota characteristics between adult patients with vitiligo (DI_a) and adult healthy controls (HC_a). **(A)** The box scatter plot displays the Chao index of the two groups; **(B)** Principal coordinate analysis (PCoA) plot is based on unweighted_unifrac distance; **(C,D)** The histograms present the composition of the top 10 intestinal microbiota at the phylum level and genus level in the two groups; **(E)** The box scatter plot demonstrates the difference in the ratio of Firmicutes/Bacteroidetes; **(F,G)** The inter-group differences in the average relative abundance of the top 10 taxa at the phylum level and genus level are depicted. FDR-adjusted *p*-values are indicated as follows: *p < 0.05; **p < 0.01; ***p < 0.001; **(H)** LEfSe analysis at the genus level revealed differential gut microbial marker genera between the two groups (LDA score > 3). **(I)** LEfSe analysis based on KEGG level 1 and level 2 functional prediction pathways (LDA score > 2).

**Figure 3:**
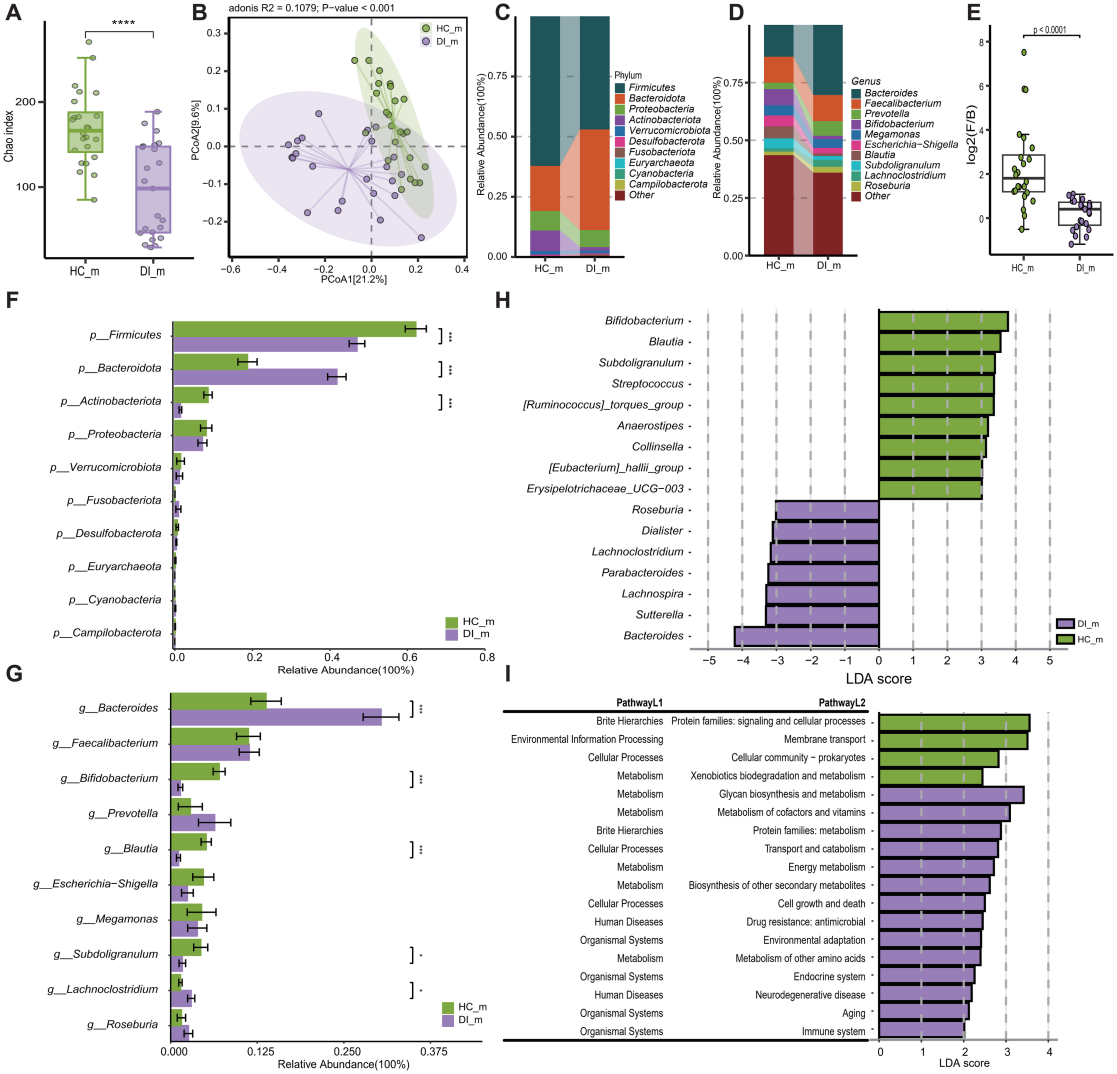
Subgroup2: Comparison of the gut microbiota characteristics between minor patients with vitiligo (DI_m) and minor healthy controls (HC_m). **(A)** The box scatter plot displays the Chao index of the two groups; **(B)** Principal coordinate analysis (PCoA) plot is based on unweighted_unifrac distance; **(C,D)** The histograms present the composition of the top 10 intestinal microbiota at the phylum level and genus level in the two groups; **(E)** The box scatter plot demonstrates the difference in the ratio of Firmicutes/Bacteroidetes; **(F,G)** The inter-group differences in the average relative abundance of the top 10 taxa at the phylum level and genus level are depicted. FDR-adjusted *p*-values are indicated as follows: *p < 0.05; **p < 0.01; ***p < 0.001; **(H)** LEfSe analysis at the genus level revealed differential gut microbial marker genera between the two groups (LDA score > 3). **(I)** LEfSe analysis based on KEGG level 1 and level 2 functional prediction pathways (LDA score > 2).

To investigate the impact of age on the gut microbiota composition of individuals with vitiligo, the study initially categorized the DI (vitiligo patients) group and HC (healthy control) group into two subgroups based on age. This stratification was conducted to assess the gut microbiota characteristics between vitiligo patients in different age categories and healthy controls in corresponding age groups. The analysis of diversity indicated that vitiligo patients, both minors and adults, exhibited notably reduced *α* diversity in their gut microbiota compared to healthy individuals (*p* < 0.05, Figures 2A, 3A). Additionally, *β* diversity showed a clear distinction between the two groups (*p* < 0.001), with a more noticeable separation observed in the minors (Figures 2B, 3B).

Disease-healthy control groups across different age groups display similar differences in microbiota composition, which correspond to the results of the overall sample grouping. These differences are mainly characterized by a rise in *Bacteroidota* and a reduction in *Firmicutes* and *Actinobacteriota* among the disease group (Figures 2C–F, 3C–F, Supplementary Table S1). However, the examination at the genus level uncovers differences in disease-related genera among different age brackets, notably highlighted by the significant enrichment of *Lachnoclostridium* in DI_m within the minors. On the other hand, *Lachnoclostridium* shows a notably higher presence in HC_a among adults, with no substantial variation between the two groups (Figures 2G, 3G, Supplementary Table S2). Additionally, it was noted that among adults, the quantity of *Bacteroides* did not significantly differ between the groups after applying the Benjamini-Hochberg correction (*p* = 0.0907, Supplementary Table S1). Nevertheless, based on Lefse analysis, *Bacteroides* was identified as a distinct genus in the disease group across different age categories. Additionally, *Parabacteroides* emerged as another prevalent indicator genus in the disease group. However, the disease-associated indicator genera, apart from these, differed between the minors and adult groups. For example, *Alistipes* and *Phascolarctobacterium* were identified as disease-associated indicator genera in the adult group, while *Roseburia, Lachnoclostridium, Dialister, Lachnospira*, and *Sutterella* were characteristic genera in the DI_m group (Supplementary Table S2). Conversely, the characteristic genera in the healthy control groups across various age groups were largely consistent, mainly including *Blautia, Subdoligranulum, Bifidobacterium, Anaerostipes, [Eubacterium]_hallii_group, Erysipelotrichaceae_UCG-003,* and *[Ruminococcus]_torques_group* (Figures 2H, 3H, Supplementary Table S2).

The outcomes of functional pathway analysis conducted with PICRUST reveal that the variations in KEGG Level 2 pathways between disease-healthy control groups of different age groups are predominantly consistent (Supplementary Table S3). Nevertheless, a more detailed investigation and comparison utilizing LEfSe unveiled that, apart from amino acid metabolism, the KEGG Level 2 pathways that exhibited differences in the adult group were also evident in the minors. Notably, the minors displayed a broader range of distinct pathways. Besides those identified in adults, the distinct pathways in minors included xenobiotic degradation and metabolism, which were significantly enriched in HC_m, along with energy metabolism, biosynthesis of various secondary metabolites, and the immune system, all notably enriched in DI_m (Figures 2I, 3I).

### Age-related differences in gut microbiota of vitiligo patients

**Figure 4:**
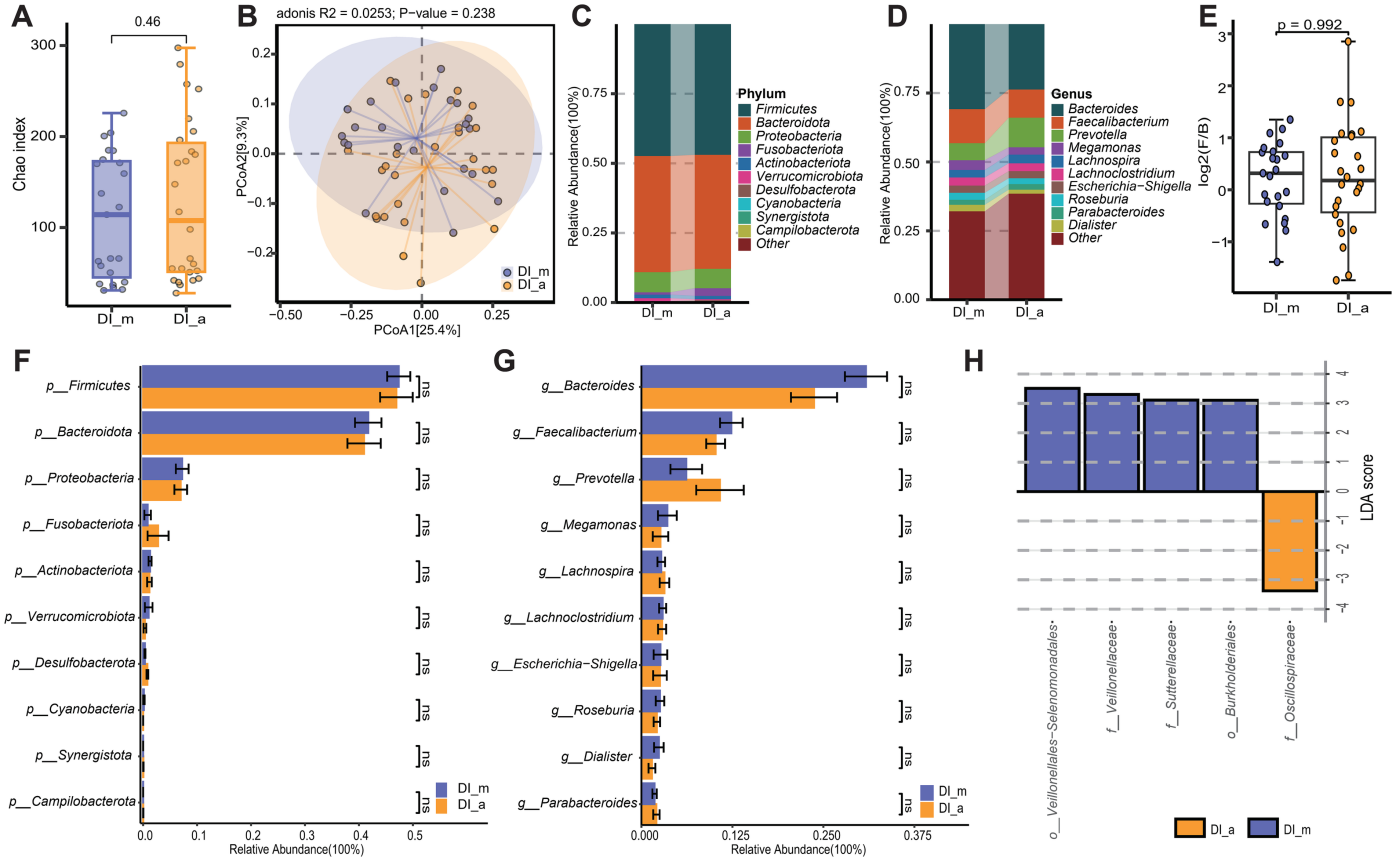
Subgroup3: Comparison of the gut microbiota characteristics between minor patients with vitiligo (DI_m) and adult patients with vitiligo (DI_a). **(A)** The box scatter plot displays the Chao index of the two groups; **(B)** Principal coordinate analysis (PCoA) plot is based on unweighted_unifrac distance; **(C,D)** The histograms present the composition of the top 10 intestinal microbiota at the phylum level and genus level in the two groups; **(E)** The box scatter plot demonstrates the difference in the ratio of Firmicutes/Bacteroidetes; **(F,G)** The inter-group differences in the average relative abundance of the top 10 taxa at the phylum level and genus level are depicted. ns, no significance; **(H)** LEfSe analysis identified variations in gut microbiota taxa between the two groups (LDA score > 3).

To investigate the impact of age on the gut microbiota of vitiligo patients, a comparative analysis was conducted between the DI_m group and the DI_a group. According to the Chao index for alpha diversity (*p* = 0.46) and the unweighted_unifrac distance for beta diversity (*p* = 0.238), the findings showed no significant statistical differences in the gut microbiota diversity between the DI_m and DI_a groups (Figures 4A,B). Examination of the main components of gut microbiota at both the phylum and genus levels showed no notable differences in taxonomic categories between the two groups (Figures 4C–G). However, using LEfSe analysis, distinct microbial biomarkers were identified at the order and family levels across different age categories of patients. Specifically, *Oscillospiraceae* was enriched in the DI_a group, while the DI_m group showed a more diverse range of microbial biomarkers, including *Veillonella, Burkholderiales, Veillonellaceae*, and *Sutterellaceae* (Figure 4H). However, the anticipated KEGG level 2 and level 3 functional pathways showed no variation between the two groups. Although these findings suggest a minimal influence of age on the gut microbiota of vitiligo patients, a series of correlation analyses were conducted to explore the potential impact of age further.

### Age-related differences in gut microbiota of healthy controls

In order to investigate the effect of age on the intestinal flora of healthy controls, we performed a comparative analysis between the HC_m and HC_a groups. Based on the Chao index of *α* diversity (*p* > 0.05) and the unweighted_unifrac distance of *β* diversity (*p* > 0.05), the results of the study showed that there was no statistically significant difference in the diversity of the gut microbiota between the HC_m and HC_a groups (Supplementary Figures S1A,B). The predominant phyla at the phylum level between the HC_m and HC_a groups were *Firmicutes*, *Bacteroidota* and *Actinobacteriota*, collectively representing over 90% of the microbiota. No significant difference was observed in the level and ratio (F/B) of the phylum Thick-walled Bacteria to the phylum *Actinobacteriota* between the two groups (Supplementary Figures S1E,F, *p* > 0.05). However, the level of *Actinobacteriota* was found to be reduced in the HC_a group in comparison to the HC_m group (Supplementary Figure S1F, *p* < 0.05). At the genus level, *Bacteroides* was the most dominant genus in both groups, and the top 10 genera in terms of relative abundance did not differ significantly between the two groups (Supplementary Figure S1G). In conclusion, no significant differences were observed in the diversity of the major gut microbiota between the HC_m and HC_a groups, with the exception of the difference in the level of *Actinobacteriota* at the phylum level.

### Correlation analysis among age, disease duration, VASI, and microbial marker genera

**Figure 5:**
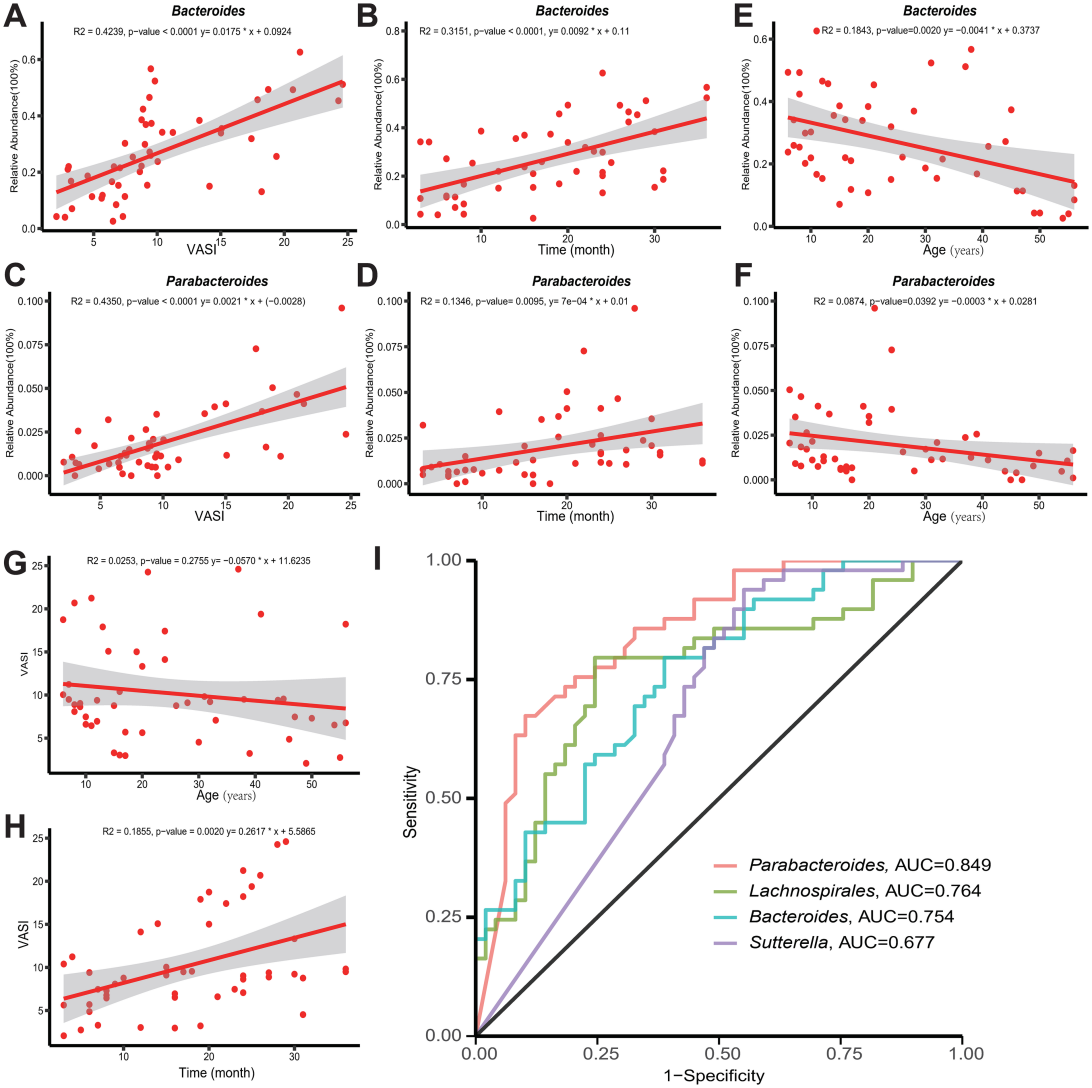
**(A,B,E)** Demonstrate the correlation between *Bacteroides* and VASI score, age, and disease duration, respectively. **(C,D,F)** Demonstrate the correlation between *Parabacteroides* and VASI score, age, and disease duration, respectively. **(G,H)** Shows the trends of VASI with age and duration of the disease, respectively. **(I)** Accuracy of *Parabacteroides*, *Lachnospira*, *Bacteroides*, and *Sutterella* as predictors of vitiligo. AUC, area under the curve; ROC, receiver operating characteristic.

Correlation analyses were conducted to investigate the association between differential marker genera related to vitiligo, specifically *Bacteroides* and *Parabacteroides*, as identified in previous results. The findings show a gradual rise in the abundance of these marker genera as the disease progresses and the VASI score increases (Figures 5A–D). Particularly, *Bacteroides* demonstrate a more pronounced pattern regarding relative abundance and trend. These findings suggest that both genera may play a significant role in the onset and progression of vitiligo, with *Bacteroides* potentially exerting a more substantial impact. However, as age increases, both genera exhibit a decreasing trend, indicating that the severity of the disease in adults may be relatively lower compared to minors with vitiligo (Figures 5E,F). Comparable outcomes were noted through the correlation analysis of VASI and age. VASI exhibited a minor decline as age increased (Figure 5G). Nonetheless, this trend was not statistically significant. Furthermore, a positive correlation is observed between the VASI score and disease duration, implying that a longer duration of illness may result in a higher VASI score (Figure 5H). In addition, ROC analyses were performed on a number of genera to assess their reliability as predictors of vitiligo, among which *Parabacteroides* (AUC = 0.849), *Lachnospira* (AUC = 0.764), *Bacteroides* (AUC = 0.754), *Sutterella* (AUC = 0.677) (Figure 5I), and ROC analyses for some of the remaining genera are shown in Supplementary Figure S2F. All four genera had some degree of diagnostic accuracy, which may help in the diagnosis of vitiligo.

## Discussion

Vitiligo is a chronic dermatological condition characterized by the specific depletion of melanocytes, with potential contributing factors including metabolic irregularities, oxidative stress, inflammation, and autoimmunity (Diotallevi et al., 2023). Recent research has underscored the significant influence of gut microbiota on host metabolism, inflammatory processes, and immune responses (Zheng et al., 2020; Fan and Pedersen, 2021). The developing idea of the gut-skin axis has linked different skin conditions to the composition of gut microbiota, yet research on the connection between gut microbiota and vitiligo is still scarce (Chen et al., 2021b; De Pessemier et al., 2021; Mahmud et al., 2022). We employed 16S rRNA sequencing to analyze the gut microbiota composition in people with stable non-segmental vitiligo, and investigated the influence of age on the condition by dividing participants into different age categories.

The gut microbiota plays a key role in immune system homeostasis by regulating the differentiation and function of immune cells, such as regulatory T cells (Tregs), maintaining immune tolerance and suppressing autoimmune responses (Round and Mazmanian, 2010). However, in patients with vitiligo, dysbiosis of the gut flora may lead to abnormal activation of immune cells, resulting in a reduced or suppressed number of Tregs and dysregulation of effector T cells (e.g., CD8+ T cells), which are more prone to attack their own tissues. Deficiencies in some gut microbiota may over-activate Th1 and Th17 cells, with Th1 secreting cytokines such as IFN-*γ* and Th17 secreting IL-17, which activate cytotoxic T-lymphocytes (CTLs), including CD8+ T cells, and further promote the attack of melanocytes by self-activated CD8+ T cells (Duan et al., 2012). Dysbiosis of the gut flora can also affect the function of antigen-presenting cells (APCs), causing changes in the way they present otherwise tolerated self-antigens. For example, certain gut bacteria can regulate the maturation and function of dendritic cells (DCs) (Macpherson and Uhr, 2004; Yang et al., 2022). Normally, APCs present antigens to T cells in a precise manner, avoiding overreaction to self-antigens. However, dysregulation of the gut microbiota may alter the expression pattern of molecules on the surface of APCs, leading to false presentation of melanocyte-specific antigens by APCs, which activates self-reactive CD8+ T cells and triggers immune attack against melanocytes. An intact gut barrier serves to prevent the penetration of harmful substances, including antigens and toxins, into the circulation. In contrast, disturbances in the gut microbiota can disrupt the gut barrier, a phenomenon known as ‘leaky gut’. This allows substances that would otherwise be confined to the gut, such as antigens, to enter the bloodstream and activate the systemic immune system (Mishra et al., 2023). These antigens may cross-react with melanocyte-derived proteins, or the activation of the immune system may indirectly lead to the activation of auto-reactive CD8+ T cells, which attack melanocytes.

Metabolites of intestinal flora have an important role in autoimmune initiation. Short-chain fatty acids (SCFAs) (acetic acid, propionic acid, butyric acid) produced by their fermented dietary fibre are critical for immune regulation, e.g., butyric acid inhibits histone deacetylase (HDAC) regulatory gene expression and affects immune cell function and differentiation (Arpaia et al., 2013). Altered production and utilization of SCFAs occurs when gut flora is dysbiotic, e.g., reduced butyrate-producing bacteria and decreased butyric acid levels in patients with inflammatory bowel disease, and this change may lead to abnormal immune cell function, making CD8+ T cells more susceptible to activation to attack melanocytes. Bile acid metabolism is closely related to intestinal flora and autoimmunity. Intestinal flora can influence the metabolic conversion of bile acids to modulate the immune response (Sayin et al., 2013; Rao et al., 2021). Disturbances in bile acid metabolism may additionally impact the immune system’s recognition of and response to self-antigens, thereby triggering autoimmunity. For instance, alterations in the intestinal flora result in aberrant bile acid metabolism, which facilitates Th17 differentiation. Th17 secretes cytokines, such as IL-17, which may activate and recruit CD8+ T cells (Duan et al., 2012), enhance the immune response to melanocyte-specific antigens, and contribute to the pathogenesis of vitiligo. The concept of the gut-skin axis emphasizes the existence of a strong immune link between the gut and the skin. Autoimmune responses initiated by intestinal dysbiosis result in the activation of immune cells and the release of cytokines, which can traverse the bloodstream from the gut to the skin. In the case of psoriasis, for instance, related inflammatory cytokines (e.g., TNF-*α* and IL-6) can reach the skin and trigger an inflammatory response. In the case of vitiligo, this may be evidenced by an increased migration of autoreactive CD8+ T cells to the skin, where they attack melanocytes and result in depigmentation. Additionally, intestinal flora may influence the skin immune environment through the neuro-endocrine pathway, regulating the production and release of neurotransmitters such as serotonin and dopamine. Serotonin is a crucial neurotransmitter for both skin function and immune regulation, and abnormal levels of serotonin may affect the response of immune cells to melanocytes.

In our investigation, a notable decline in the alpha diversity of gut microbiota was observed in individuals with vitiligo in comparison to healthy controls. This pattern remained consistent across analyses conducted on the entire sample and in subgroup analyses based on age. The results indicate that individuals with vitiligo demonstrate reduced diversity in their gut microbiota, which is in line with recent studies reported by Bzioueche et al. (2021) and Luan et al. (2023). However, Ni et al. (2020) reported an increase in gut microbiota diversity in vitiligo patients, but Principal Component Analysis (PCA) contributed the most with 24.53%, also suggesting differences in gut community structure in vitiligo patients. While there are a limited number of conflicting reports, this may be attributed to the scarcity of studies conducted on the subject. The prevailing body of current research consistently upholds the notion of gut microecological dysregulation and reduced diversity in various disease conditions (De Pessemier et al., 2021; Afzaal et al., 2022; Hou et al., 2022). This phenomenon is observed not only in skin disorders like acne vulgaris, atopic dermatitis, and psoriasis but also in other systemic illnesses. Consistent with previous research, the *β*-diversity outcomes derived from Principal Coordinate Analysis (PCoA) effectively differentiate between individuals and healthy controls, emphasizing the spatial heterogeneity in gut microbiota among vitiligo patients and healthy individuals (Ni et al., 2020; Wu et al., 2023). Nevertheless, no distinction was evident between the DI_m and DI_a groups stratified by age. Generally, the gut microbiota diversity in healthy children differs from that in adults (Radjabzadeh et al., 2020; Korpela et al., 2021). Research on healthy children in northwestern China revealed notable alterations in their gut microbiome as they aged. At the phylum level, the abundances of *Actinobacteria* notably decreased as children aged. At the genus level, the abundances of *Eggerthella, Erysipelatoclostridium, Tyzzerella, Flavonifractor*, and *Bididobacterium* also significantly declined with age, whereas *Coprobacer, Lactobacillus, Oscillibacter, Odoribacter* and *Christensenella* showed a marked increase as children grew older (Gao et al., 2023). In our results, there was also a decrease in *Actinobacteriota* in the HC_a group compared to the HC_m group at the phylum level, but we did not find a significant change at the genus level. Whether the disease state weakens the influence of age remains unknown, particularly in immune system-related skin disorders like vitiligo, necessitating further investigation.

In the microbiome composition, *Firmicutes* and *Bacteroidota* are identified as crucial components at the phylum level in both diseased and healthy control groups, consistent with previous research (Bzioueche et al., 2021; Luan et al., 2023; Wu et al., 2023). Notably, our analysis unveiled a marked decrease in the *Firmicutes/Bacteroidota* ratio within the vitiligo group, contrasting the findings of Bzioueche et al. (2021) and Wu et al. (2023). Additionally, recent research by Luan et al. (2023) did not detect significant variations in this ratio between the groups under comparison. These contradictory findings underscore the necessity for further exploration into assessing the F/B ratio in the gut microbiota of vitiligo patients. Remarkably, genera linked to butyrate production, such as *Bifidobacterium, Blautia, Subdoligranulum,* and *Agathobacter,* exhibited substantial decreases in the vitiligo cohort. Butyrate, a crucial SCFA, not in reducing systemic inflammatory responses by preserving the intestinal epithelial barrier but also participates in the citric acid cycle to lower mitochondrial reactive oxygen species production, thereby alleviating autoimmune responses triggered by oxidative stress (Donohoe et al., 2011; Xiao et al., 2022; Xie et al., 2016). Furthermore, SCFA have the potential to impact the activity and quantity of Tregs, which could play a role in the pathogenesis and advancement of vitiligo (Smith et al., 2013; Jin et al., 2024). The genus *Bacteroides* is significantly enriched in the disease group and is also an important genus that produces short-chain fatty acids (Cheng et al., 2022). Nevertheless, this genus influences the host in multiple ways, impacting not just autoimmune diseases but also affecting the onset and development of cancer via intricate biological processes (Zafar and Saier, 2021; Shin et al., 2024). Contradictory to previous studies, Bzioueche et al. (2021) have reported a decrease in the prevalence of *Bacteroides* in individuals with vitiligo. This variation may be attributed to the specificity of different *Bacteroides* species, as there are significant differences among them. For instance, while *Bacteroides fragilis*, *Bacteroides plebeius*, and *Bacteroides uniformis* are notably reduced in individuals with chronic urticaria, *Bacteroides ovatus* and *Bacteroides uniformis* are elevated in patients with atopic dermatitis (Lu et al., 2019; Ye et al., 2021). Similarly, the most recent results from Luan et al. (2023), based on metagenomic sequencing, indicate that *Bacteroides fragilis* is enriched in the vitiligo patient group, while *Bacteroides bouchesdurhonensis* is reduced. In light of these findings, we postulate that this may be the underlying reason for the discrepancies observed in *Bacteroides* results across several recent studies on vitiligo-related intestinal microbiota. Consequently, future studies should prioritize metagenomic analysis strategies that are accurate to the species or even strain level.

When analyzed using LEfSe, it was found that *Oscillospiraceae* was enriched in the DI_a group. *Oscillospiraceae*, a family of microbes commonly found in the intestines of both humans and animals, have their *Oscillospira* levels affected by numerous elements such as age, dietary habits, prebiotics, probiotics, and exposure to heavy metals. An increase in *Oscillospira* abundance was linked to a high-fat diet, obesity, type 2 diabetes, constipation, and depression, while the trembling spirochete showed a direct connection to gallstones and could serve as a biomarker for symptomatic gallstone formation. The presence of Oscillospira was notably lower in individuals suffering from inflammatory bowel disease and showed a negative correlation with the intensity of enteritis and enteropathy (Yang et al., 2021). *Oscillospira* abundance was notably lower in the gut of overweight children (Chen et al., 2021c). Aging involves a persistent, mild inflammation marked by increased levels of inflammatory substances in the bloodstream. Circulating concentrations of many inflammatory markers and mediators are higher in older adults than in young adults. Increasing research indicates that age-related dysregulation of the gut microbiota contributes to the overall inflammatory state in older adults (Shintouo et al., 2020). Among these, *Oscillospira* is strongly negatively correlated with pro-inflammatory monocyte chemotactic protein 1 (Conley et al., 2016). *Oscillospira* levels rise as people get older, but they decline in some age-related illnesses or conditions linked to poor aging, such as cardiometabolic diseases (Taniguchi et al., 2018). Variations in the microbiome due to aging are significantly diverse and shaped by personal and external environmental influences. Ageing is accompanied by changes in the microbiome, which in turn affects the rate of age-related decline (Ghosh et al., 2022). Microbial biomarkers in the DI_m group were more diverse and included *Veillonella, Burkholderiales, Veillonellaceae and Sutterellaceae*. *Veillonella* are bacteria that significantly contribute to various bodily regions, such as the oral cavity, gut, and respiratory system. The impact of microorganisms of the genus *Veillonella* varies significantly in different populations, especially in children and the elderly. In children, *Veillonella parvula* might play a protective role in the initial stages of immune system development, potentially aiding in the prevention of asthma, bronchitis, and autism (Arrieta et al., 2015; Djais et al., 2019). Research in epidemiology indicates that the abundance of *Veillonella parvula*, commonly found in the gut, is markedly reduced in children who are susceptible to asthma. In addition, *Veillonella* plays an important role in children’s oral health, acting as an early settling species of oral biofilms, forming aggregates with a wide range of bacteria, providing nutrients for periodontal pathogens, and influencing oral ecology (Djais et al., 2019). In adults, *Veillonella’s* impact is then associated with a variety of diseases. For instance, the abundance of *Veillonella* is notably higher in conditions like primary sclerosing cholangitis, non-alcoholic fatty liver disease, and non-alcoholic steatohepatitis (Kummen et al., 2017; Loomba et al., 2020). In addition, *Veillonella atypica* was more enriched in athletes because of its ability to utilize lactic acid as a carbon source to convert lactic acid into propionic acid, improving athletic performance and endurance (Scheiman et al., 2019). *Burkholderiales* is an order containing a wide variety of bacteria, some species of which are directly related to human health (respiratory infections, chronic lung diseases). *Sutterellaceae* is a group of bacteria found in the human gut that includes the genera *Parasutterella* and *Sutterella*. *Parabacteroides distasonis* is a bacterium in the family *Sutterellaceae* and is considered one of the core flora of the human body. There was a strong inverse relationship with conditions like obesity, non-alcoholic fatty liver disease, and diabetes, indicating it might positively influence glycolipid metabolism. This bacterium has the potential to alleviate obesity symptoms, insulin resistance, lipid metabolism issues, and NAFLD by producing succinic acid and secondary bile acids, thereby activating various signaling pathways and serving as a multi-targeted regulator. It is considered a promising probiotic for combating metabolic syndrome (Wang et al., 2019). Research discovered that *Sutterella* produced excessive IgA protease, breaking down IgA and thus reducing its levels in the gut lining, which weakened the intestinal antimicrobial immune defense (Kaakoush, 2020), and could be linked to immune irregularities in vitiligo. In summary, microorganisms of the genus *Oscillospiraceae* and *Veillonella* have diverse roles and influences in different populations, and their protective effects in children differ from the potential risks associated with disease in adults. Future studies need to further explore the specific mechanisms of action and health effects of microorganisms of the genera *Oscillospiraceae* and *Veillonella* in different populations. Based on the LEfSe analysis, it was elucidated that the characteristic microbiota in vitiligo patients is predominantly characterized by *Bacteroides*, whereas the characteristic microbiota in the healthy control group is primarily dominated by *Bifidobacterium*. *Bifidobacterium*, a renowned probiotic, produces short-chain fatty acids like butyrate and influences tryptophan metabolism, thereby improving atopic dermatitis via the gut-skin connection (Fang et al., 2022). Intriguingly, Bzioueche et al. (2021) observed a depletion of both *Bifidobacterium* and *Bacteroides* in the lesional skin microbiota of vitiligo patients. Consequently, further investigation is needed to explore the relationship between gut microbiota and skin microbiota. Moreover, subgroup analysis conducted on disease-healthy controls also revealed similar distinct outcomes. In addition to the aforementioned major differential genera, *Parabacteroides*, another distinct genus, was identified in the disease group through subgroup analysis. Analogous to *Bacteroides*, this genus not only generates short-chain fatty acids like acetic acid but also serves as an important bacterial source of sphingolipids, which can modulate immunity and inflammation by stimulating natural killer T cells and triggering rapid cytokine release (Heaver et al., 2018; Lei et al., 2021a; Cui et al., 2022). Consistent with our study, Liu et al. (2024) recently observed an enrichment of both genera in infants with severe atopic dermatitis. However, in a vitiligo-related study by Wu et al. (2023), contradictory results were reported. As mentioned earlier, these discrepant results may be limited not only by species specificity but also by limited studies.

The analysis of predicted functional pathways highlights the significance of glycan biosynthesis and metabolism, as well as the metabolism of cofactors and vitamins pathways in disease groups, and the Hypoxia-inducible factor 1 (HIF-1) pathway. Glycans play a crucial role in cellular biology, and any changes in their biosynthesis and metabolism can have implications for disease mechanisms such as autoimmunity and chronic inflammation (Alves et al., 2022; Pinho et al., 2023). In a recent study, Ali et al. (2022) discovered a notably higher presence of advanced glycation end-products (AGEs) in the affected skin of individuals with vitiligo when contrasted with healthy subjects. Additionally, vitamins are essential for maintaining intestinal immune homeostasis, which involves antibody production and the activation of immune cells (Hosomi and Kunisawa, 2017). For instance, the active variant of 1α,25-dihydroxyvitamin D3 plays a role in regulating immune reactions by influencing the function of immune cells such as T cells and B cells (Mann et al., 2015; Hosomi and Kunisawa, 2017). A recent study by Agarwal et al. (2015) indicated that the average serum levels of vitamin B12 in vitiligo patients were notably lower than those in the control group. Nonetheless, it is important to highlight that vitamin C, recognized for its antioxidant and immune-regulating properties, is not recommended for the treatment of vitiligo, as it may disrupt melanin production pathways (Chen et al., 2021a; Dutta et al., 2022). In addition, Metabolic processes, such as the fermentation of dietary fibre by intestinal flora to produce SCFAs, consume oxygen, thereby reducing the local oxygen concentration in the intestine and creating a hypoxic microenvironment. This hypoxic state will activate the intracellular hypoxia-sensing mechanism, which will stabilize and activate HIF-1, a key transcription factor that regulates a series of genes related to hypoxia adaptation. These include genes promoting angiogenesis, altered glucose metabolism and cell proliferation and survival, which enable the cells to adapt to the hypoxic environment. The activation of the HIF-1 pathway will consequently impact the metabolism and function of intestinal epithelial cells, thereby modifying the intestinal microenvironment, including pH and redox status. These modifications will influence the survival and reproduction of the intestinal flora, resulting in an increase or decrease in the number of specific flora, which in turn affects the composition of the intestinal flora. Additionally, the HIF-1 pathway can regulate the absorption and transport of certain nutrients by the intestinal epithelial cells, which may indirectly affect the metabolic activities of the intestinal flora. To illustrate, the fermentation and utilization of substances such as dietary fibre by the intestinal flora may be affected, resulting in alterations to the production of metabolites by the intestinal flora (Pral et al., 2021). Modifications to the structure of the flora and its metabolites may influence the local or systemic immune response of the body, thereby affecting the development of vitiligo.

Finally, we investigated the potential relationship between age and vitiligo characteristics based on microbiota and disease severity. The findings revealed a positive association between *Bacteroides* and *Parabacteroides* with the VASI score and disease duration in vitiligo patients. Nevertheless, as the patients aged, a downward trend was observed. Moreover, an upward trend was observed in VASI concerning disease duration, although no significant correlation was found with age. This finding is similar to the conclusions drawn by Demirkan et al. (2018), who observed that VASI in individuals with vitiligo showed a moderate association with the disease duration (*r* = 0.555; *p* < 0.001) and a weaker correlation with age (*r* = 0.349; *p* = 0.043). Nevertheless, these results contradict those reported by Patvekar et al. (2017). Given the scarcity of pertinent research available at present, additional exploration is required. Furthermore, some studies have indicated a notable positive correlation among VASI, disease duration, lesion location, and quality of life index (Algharably et al., 2019; Awal et al., 2024). However, our study did not incorporate other pertinent indicators, which constitutes a limitation of this research. In addition, we evaluated a number of genera as predictors of vitiligo, among which *Parabacteroides*, *Lachnospira*, *Bacteroides*, and *Sutterella* had some degree of diagnostic accuracy, which may help in the diagnosis of vitiligo.

Currently, some studies have revealed the efficacy of probiotics in the treatment of certain skin disorders such as atopic dermatitis, acne and psoriasis (Szántó et al., 2019). Researchers are actively exploring the relationship between microbiome changes and the development of vitiligo in order to identify potential therapeutic targets. Touni et al. (2024b) found that oral administration of neomycin affected the gut microbiota, thereby delaying the development of vitiligo in mice. A recent report showed that *Bacillus subtilis*-derived-exopolysaccharide halts depigmentation and autoimmunity in vitiligo (Touni et al., 2024a). The preceding studies indicate that the utilization of microbiome-targeted therapies has the potential to serve as a valuable strategy for preserving overall skin health and managing various dermatological conditions. While the application of microbiome-targeted therapies for vitiligo remains to be extensively researched, the modification of the microbiome composition through probiotics or analogous medications has the potential to emerge as an effective alternative approach for treating vitiligo in the future. This approach has the potential to mitigate the adverse effects associated with conventional therapies and enhance the therapeutic efficacy of the drugs, thereby reducing symptoms and improving the quality of life of patients with vitiligo.

In conclusion, the research findings not only illustrate variations in the composition of gut microbiota between vitiligo patients and healthy individuals within the same age group but also emphasize the age-related aspect of these variances. The study identifies specific microbial markers in the gut microbiota of vitiligo patients across different age groups. However, further investigation is crucial in the field of age-related disease microbiome studies. Upcoming studies ought to utilize sophisticated methods like metagenomics and metabolomics to deeply investigate the variations in gut microbiota at a finer species and metabolite functional level concerning disease onset and advancement. Additionally, increasing the sample size and conducting multicenter studies are essential to validate the reliability of the results. These endeavors will establish a theoretical basis for advancing the clinical application of gut microbiota-based therapeutic interventions for vitiligo.

## Data Availability

The datasets presented in this study can be found in online repositories. The names of the repository/repositories and accession number(s) can be found at: https://ngdc.cncb.ac.cn/gsa/s/H0LO6PGh, CRA018232.

## Supplementary material

SUPPLEMENTARY FIGURE S1 Subgroup4: Comparison of the gut microbiota characteristics between minor healthy controls (HC_m) and adult healthy controls (HC_a). **(A)** The box scatter plot displays the Chao index of the two groups; **(B)** Principal coordinate analysis (PCoA) plot is based on unweighted_unifrac distance; **(C,D)** The histograms present the composition of the top 10 intestinal microbiota at the phylum level and genus level in the two groups; **(E)** The box scatter plot demonstrates the difference in the ratio of Firmicutes/Bacteroidetes; **(F,G)** The inter-group differences in the average relative abundance of the top 10 taxa at the phylum level and genus level are depicted. FDR-adjusted *p*-values are indicated as follows: **p* < 0.05; ***p* < 0.01; ns, no significance; **(H)** LEfSe analysis identified variations in gut microbiota taxa between the two groups (LDA score > 3); **(I)** LEfSe analysis based on KEGG level 1 and level 2 functional prediction pathways (LDA score > 2).

SUPPLEMENTARY FIGURE S2 **(A–E)** The comparison of enrichment levels of HIF-1 signalling pathway in each group. healthy controls (HC); minor healthy controls (HC_m); adult healthy controls (HC_a); patients with vitiligo (DI); minor patients with vitiligo (DI_m); adult patients with vitiligo (DI_a). (**p* < 0.05; ***p* < 0.01; ****p* < 0.001). **(F)** Reliability of ROC analysis for predicting gut flora as a predictor of vitiligo. AUC, area under the curve; ROC, receiver operating characteristic.
