# Supplementary figures and images for: Identification of disease-specific gut microbial markers in vitiligo
Source: Front Microbiol. 2025 Feb 4;16:1499035. doi: 10.3389/fmicb.2025.1499035 (PMC11833150; doi:10.3389/fmicb.2025.1499035)

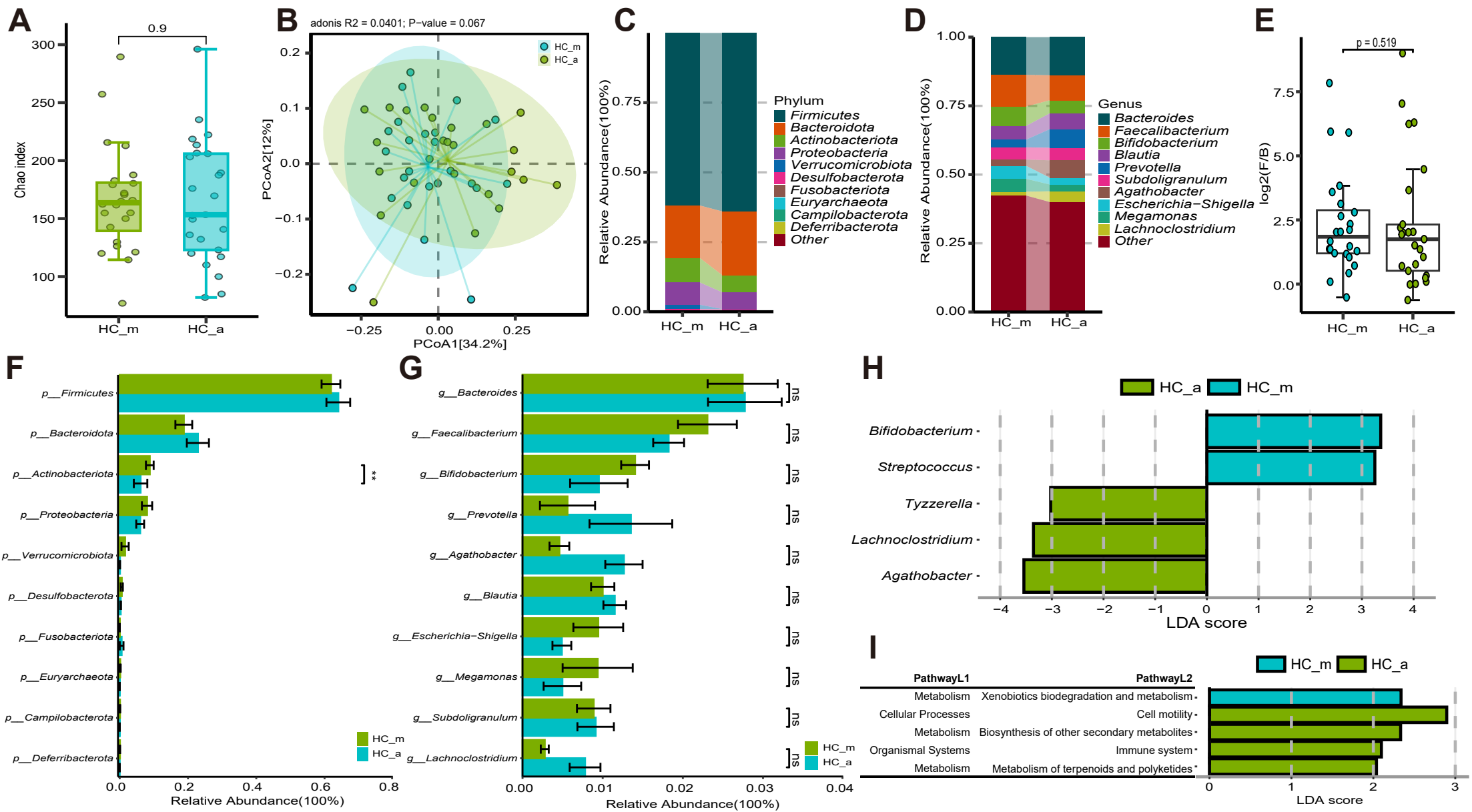

Supplement: SUPPLEMENTARY FIGURE S1 — Subgroup4: Comparison of the gut microbiota characteristics between minor healthy controls (HC_m) and adult healthy controls (HC_a). (A) The box scatter plot displays the Chao index of the two groups; (B) Principal coordinate analysis (PCoA) plot is based on unweighted_unifrac distance; (C,D) The histograms present the composition of the top 10 intestinal microbiota at the phylum level and genus level in the two groups; (E) The box scatter plot demonstrates the difference in the ratio of Firmicutes/Bacteroidetes; (F,G) The inter-group differences in the average relative abundance of the top 10 taxa at the phylum level and genus level are depicted. FDR-adjusted p-values are indicated as follows: *p < 0.05; **p < 0.01; ns, no significance; (H) LEfSe analysis identified variations in gut microbiota taxa between the two groups (LDA score > 3); (I) LEfSe analysis based on KEGG level 1 and level 2 functional prediction pathways (LDA score > 2). [file Data_Sheet_1.zip › Supplementary Figure 1.PDF]

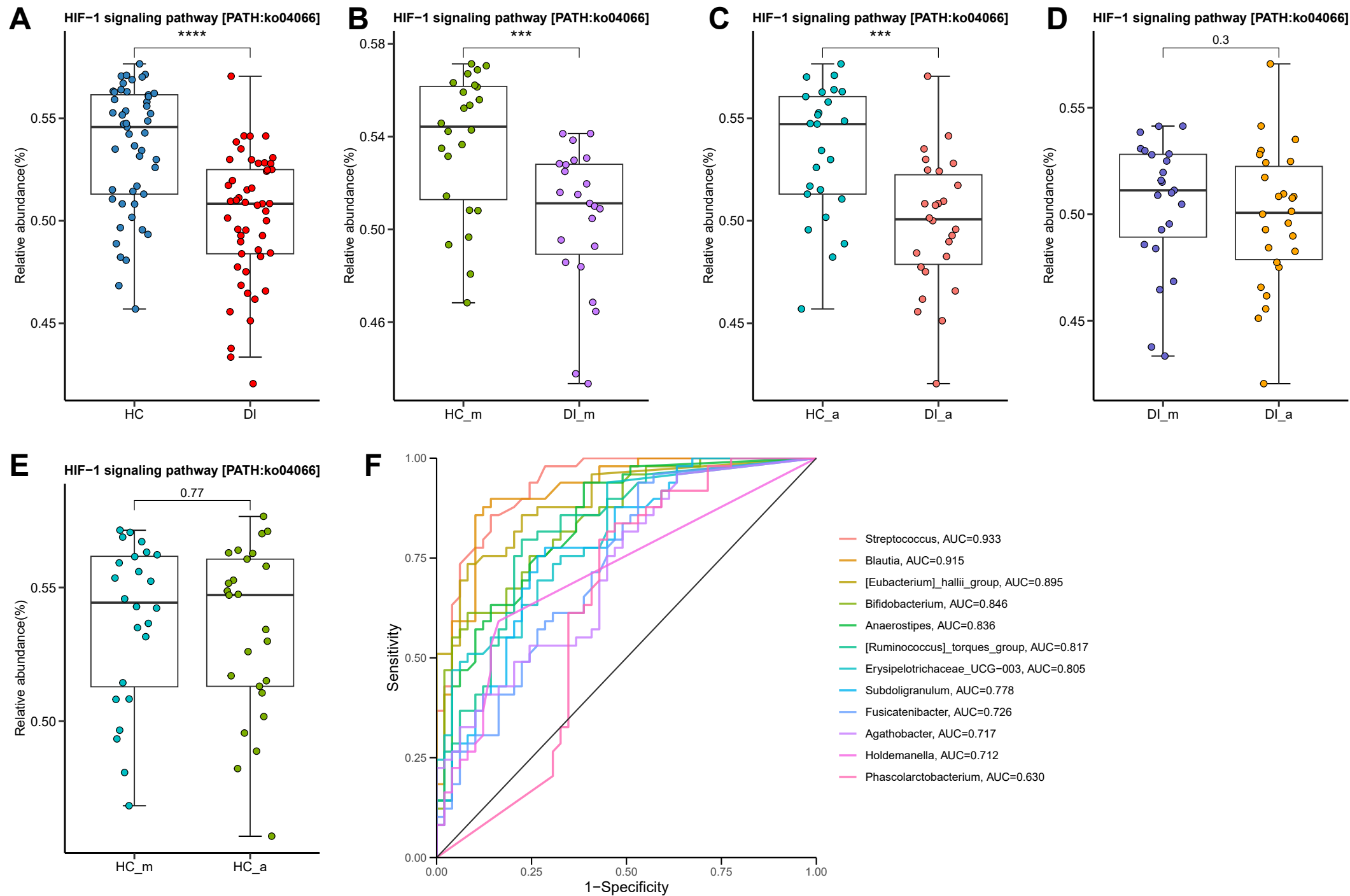

Supplement: SUPPLEMENTARY FIGURE S1 — Subgroup4: Comparison of the gut microbiota characteristics between minor healthy controls (HC_m) and adult healthy controls (HC_a). (A) The box scatter plot displays the Chao index of the two groups; (B) Principal coordinate analysis (PCoA) plot is based on unweighted_unifrac distance; (C,D) The histograms present the composition of the top 10 intestinal microbiota at the phylum level and genus level in the two groups; (E) The box scatter plot demonstrates the difference in the ratio of Firmicutes/Bacteroidetes; (F,G) The inter-group differences in the average relative abundance of the top 10 taxa at the phylum level and genus level are depicted. FDR-adjusted p-values are indicated as follows: *p < 0.05; **p < 0.01; ns, no significance; (H) LEfSe analysis identified variations in gut microbiota taxa between the two groups (LDA score > 3); (I) LEfSe analysis based on KEGG level 1 and level 2 functional prediction pathways (LDA score > 2). [file Data_Sheet_1.zip › Supplementary Figure 2.PDF]
